# Efficacy of Systematic Early-Second-Trimester Ultrasound Screening for Facial Anomalies: A Comparison between Prenatal Ultrasound and Postmortem Findings

**DOI:** 10.3390/jcm12165365

**Published:** 2023-08-18

**Authors:** Bruno Lamanna, Miriam Dellino, Eliano Cascardi, Mia Rooke-Ley, Marina Vinciguerra, Gerardo Cazzato, Antonio Malvasi, Amerigo Vitagliano, Pierpaolo Nicolì, Michele Di Cosola, Andrea Ballini, Ettore Cicinelli, Antonella Vimercati

**Affiliations:** 1 Department of Biomedical Sciences and Human Oncology, University of Bari “Aldo Moro”, 70121 Bari, Italy; 2 Fetal Medicine Research Institute, King’s College Hospital, London SE5 9RS, UK; 3 Department of Medical Sciences, University of Turin, 10124 Turin, Italy; 4 Pathology Unit, FPO-IRCCS Candiolo Cancer Institute, 10060 Candiolo, Italy; 5 Department of Precision and Regenerative Medicine and Jonic Area, University of Bari “Aldo Moro”, 70121 Bari, Italy; 6 Department of Clinical and Experimental Medicine, University of Foggia, Via Rovelli 50, 71122 Foggia, Italy; 7 Department of Precision Medicine, University of Campania “Luigi Vanvitelli”, 80138 Naples, Italy

**Keywords:** prenatal ultrasound, fetal face anomalies, cleft lip and palate, micrognathia, retrognathia, anophthalmia, autopsy

## Abstract

Second-trimester 2D ultrasound (US) assessment of the fetal anatomy, as proposed by worldwide guidelines, allows detecting the majority of fetal malformation. However, the detection rates of fetal facial anomalies seem to still be low, mostly in cases of isolated facial malformation. The purpose of this research was to assess and analyze the concordance between the antenatal imaging findings from second-trimester US screening and the results of fetal postmortem autopsy. Between January 2010 and January 2020, there were 43 cases where fetuses with prenatal ultrasound diagnosis of a face abnormality, associated or not with a genetic syndrome or chromosomal disorder, following intrauterine death (IUD) or termination of pregnancy (TOP) after the 13 weeks of pregnancy, underwent autopsy in the Pathological Anatomy section of Bari Polyclinic specializing in feto-placental autopsies. The diagnosis of the fetal facial defects at ultrasound was compared with the findings at autopsy in all cases. A very high level of agreement between prenatal ultrasound and autopsy findings was found for facial abnormalities associated with genetic syndromes or numerical abnormality of chromosomes. A lower level of concordance was instead found in isolated facial defects or those associated with other organ anomalies, but not associated with genetic syndrome or numerical chromosome anomaly. A detailed examination of aborted fetuses led to successful quality control of early-second-trimester ultrasound detection of facial anomalies; however, it was less accurate for the isolated ones. It is, thus, reasonable to propose a systematic early-second-trimester prenatal ultrasound screening for facial anatomy by operators specialized in fetal medicine field, using 2D, 3D, and 4D techniques (two-, three-, and four-dimensional ultrasound).

## 1. Introduction

The prenatal diagnosis of craniofacial abnormalities presents unique challenges due to the wide range of morphological features involved. However, significant improvements in ultrasound (US) and magnetic resonance imaging (MRI) techniques have enabled the recognition of facial anomalies before birth, which were previously only detectable after delivery. This advancement allows for better parental counseling, evaluation of associated genetic causes, and careful planning of delivery and postnatal treatment. Despite these advancements, US accuracy may still be limited by various factors, such as maternal body habitus, early gestational age, complex fetal anomalies, fetal position, oligohydramnios (reduced amniotic fluid), and operator expertise [1].

Early assessment of the fetal facial anatomy can be conducted during the early stages of pregnancy. From the 12th week of gestation, different parts of the fetal facial anatomy, such as the orbits, nose, and forehead, can be identified. As pregnancy progresses beyond the 16th week, a more detailed assessment of the forehead, nose, lips, ears, and orbits of the fetus becomes possible.

The ultrasonic detection [2] of fetal facial abnormalities must be considered part of a wider and more complex prenatal investigation path, in which the ultrasound finding can be highly suggestive of a fetal structural anomaly, but not exhaustive for the diagnosis of multiple genetic syndromes. Multidisciplinary counseling with a gynecologist, geneticist, and pediatrician has to be proposed to the pregnant woman and the partner in order to discuss the possible association between facial anomalies and multiple genetic syndromes, enabling the parent to decide whether to eventually continue with further diagnostic tests such as chromosomal analyses. The results obtained can affect the decision of the mother regarding the continuation of the pregnancy. Moreover, it can help the physician to determine the timing, method, and birth center for delivery and post-birth management in order to guarantee good perinatal outcomes. Facial abnormalities can affect the nose, eye sockets, lip, jaw, or palate.

According to the ISUOG practice guidelines for mid-trimester fetal ultrasound scan, the minimum requirements for routine fetal face evaluation include a transverse scan of both orbits and a coronal scan of the nose/nostril, mouth, and mostly upper lip integrity. If it is technically feasible, an evaluation of the median facial profile is also highly suggested, which is useful for the diagnosis of cleft lip, micrognathia, and nasal bone anomalies. The only approach proposed is the 2D US technique [3]. In agreement with the emerging need to provide new tools for earlier prenatal diagnosis, new recommendations were defined in 2023 by the ISUOG Practice Guidelines for the performance of the 11–14-week ultrasound scan. The early-second-trimester visualization of the fetal face should be achieved through a 2D approach and in the midsagittal plane, which should be complemented with examination in either the axial or the coronal plane. The magnified midsagittal plane of the head and neck enables the assessment of several anatomic regions of the face, including the forehead, nasal bone, maxilla, mandible, and mouth. Different facial angles and markers (e.g., maxillary gap, superimposed line sign) have been proposed to assess the presence of facial clefts in the midsagittal view, but these need confirmation in other planes. In an axial or coronal view, an attempt should be made to visualize the eyes with their interorbital distance and the retronasal triangle, demonstrating the maxilla and the mandible. The nasal bone being ‘absent’ or hypoplastic, in addition to the anatomical findings, can be used as a marker to improve the efficacy of ultrasound-based screening for T21 [4]. Despite the exclusive 2D approach proposed in the routine study of facial anatomy by the aforementioned guidelines. The fetal face can be evaluated using 2D, 3D, and 4D ultrasound (two-, three-, and four-dimensional ultrasound) [5]. With 2D, the fetal face can be studied on three planes: sagittal, transverse (axial), and coronal. With the sagittal plane, we can detect dysmorphism of the forehead and nose, thus enabling detection of the nasal bone and chin (micrognathia/retrognathia). The axial plane, on the other hand, allows assessment from the orbits with the binocular distance, the interocular distance, and the ocular diameter. Descending caudally along the fetal head, it is possible to evaluate the upper lip with the palate, as well as the dental buds, the mandible, and the maxilla. The coronal plane is essential for the evaluation of the mouth, lips, and nose. The 3D ultrasound is an important supplementary tool for 2D ultrasound in the spatial evaluation of the fetal face and its anomalies. The 4D ultrasound adds a dynamic component to the 3D exam, which makes it possible to evaluate certain fetal expressions.

### 1.1. Eyes

The development of the eyeball is completed within 40 days after conception, with subsequent differentiation of the lens and ocular structures [6]. From the late first trimester, we should consider the visualization of the fetal orbit and lens. The orbits appear as echogenic circles on the upper side of the fetus, while the lens can be visualized inside these structures as circular hyperechogenic rings. The coronal and particularly axial planes of the fetal head are the best approach for assessing fetal eyes. Some authors published tables of normal orbital distance values in relation to gestational age. In utero evaluation of the interlens distance is important, as it expands our knowledge of the development of the fetal eye and any associated genetic abnormalities. Hypertelorism is an interocular distance above the 95th percentile and can be associated with chromosomal defects, mainly trisomy 13. Genetic syndromes are found in >50% of cases. The most common are frontonasal dysplasia, craniosynostosis, and Neu–Laxova syndrome. Hypertelorism can be associated with encephalocele and agenesis of the corpus callosum [7]. The accuracy of the ultrasound exam in hypertelorism diagnosis has not been established. Hypotelorism is a decreased interorbital diameter <5th centile and is among the migration defects together with holoprosencephaly. The degree of hypotelorism can be extreme as in cyclopia. Hypotelorism can be associated with chromosomal abnormalities, mainly trisomy 13. Genetic syndromes are very frequent, and the most common is Meckel–Gruber syndrome [8].

#### 1.1.1. Microphthalmia and Anophthalmia

Microphthalmia is defined as a small size of the eye diameter below the fifth percentile for gestational age while anophthalmia represents the absence of ocular structures. Microphthalmia and anophthalmia can be unilateral or bilateral. In the case of microphthalmia/anophthalmia, a thorough ultrasound examination should be conducted in order to exclude associated defects (microtia, micrognathia, syndactyly, camptodactyly, median cleft, feet abnormalities, hemivertebrae, and congenital heart defects), as well as invasive testing for karyotyping and array CGH (microarray-based comparative genomic hybridization). Both anophthalmia and microphthalmia may occur in isolation or as part of a syndrome. Genetic causes include chromosomal abnormalities (Patau syndrome, mosaic trisomy 9, 13q deletion syndrome) or monogenetic Mendelian disorders (CHARGE syndrome, Fraser syndrome, oculofaciocardiodental syndrome) [9]. Some environmental and maternal risk factors have been implicated as causes of microphthalmia and anophthalmia, either in isolation or in combination with genetic causes. Examples include alcohol consumption during pregnancy, maternal exposure to teratogenic medications (e.g., isotretinoin, warfarin, nitrofurantoin, or thalidomide), maternal womb infections (e.g., rubella virus, CMV, or influenza), maternal age over 40 years old, infants with low birthweight (less than 2.5 kg), and maternal vitamin A deficiency [10].

#### 1.1.2. Cataracts

Congenital cataracts are a rare occurrence, affecting approximately 1 to 6 out of 10,000 live births. Prenatal diagnosis of this condition involves the visualization of an extremely echogenic lens. It is worth noting that, in 30% of cases, congenital cataracts are unilateral, while, in 50% of cases, they present bilaterally.

Genetic syndromes are observed in about 10% of congenital cataract cases, with common examples including Walker–Warburg (autosomal recessive) and chondrodysplasia punctata (X-linked recessive). Additionally, cataracts are associated with congenital infections in around 30% of cases, especially linked to infections such as rubella, toxoplasmosis, and CMV [11].

#### 1.1.3. Cyclopia

Cyclopia is a rare type of holoprosencephaly and congenital disorder characterized by the failure of the embryonic brain to correctly divide the eye orbits into two cavities. The eye is placed at the center of the area, usually occupied by the root of the nose. Typically, there is either a missing nose or a non-functional nose in the form of a proboscis (a tubular appendix) above the central eye. Cyclopia occurs in 1 in 16,000 live births [12]. Cyclopia is often associated with genetic syndromes such as trisomy 18 (Edwards syndrome) or trisomy 13 (Patau syndrome). Risk factors associated with cyclopia include fetal factors (monozygotic twins with one twin normal and the other with cyclopia chromosomal abnormalities) and maternal factors (drugs during pregnancy, consanguineous marriages, ultraviolet light, gestational diabetes) [13]. Axial and sagittal fetal scans allow diagnosis and viewing of the proboscis.

#### 1.1.4. Dacryocystocele

Dacryocystocele is caused by obstruction of the lacrimal drainage system, which in turn causes dilation of the nasolacrimal sac [14]. Dacryocystocele is mostly a transient finding; it may resolve spontaneously in utero or postnatally with warm and wet compresses or surgery at birth [14]. Axial and sagittal fetal scans allow one to visualize unilateral or bilateral cysts between the lower part of the orbit and the nose.

### 1.2. Ears

Ear development starts in the third week of conception and generally ends in the 20th week [15]. Ear anomalies may include anotia, microtia, macrotia, and abnormal position or shape. Conditions associated with ear anomalies include trisomies (13, 18, 21) Turner syndrome, holoprosencephaly, Crouzon syndrome, Treacher Collins syndrome, choanal atresia, and VACTERL syndrome. Prenatal diagnosis of ear anomalies is also possible with 2D and 3D examination, although we are now aware that 3D is more accurate. Chang and co-authors demonstrated how 3D ultrasound is able to assess the shape of the fetal ear in 93% of cases compared to 40% for 2D ultrasound. In addition to ultrasound technology, fetal MRI can be useful in studying the ear. Low-set ears are typical features of Turner syndrome [16].

### 1.3. Nose and Nostrils

The median sagittal section of the fetal face is ideal for detecting the nasal bone from the 11th week. The upper line is represented by the skin, while the lower line, thicker and more echogenic than the skin above, represents the nasal bone. Ultrasound visualization of the nasal bone is part of first-trimester fetal screening, with increased sensitivity today. The absence of ossification of two bilateral nasal bones constitutes an additional marker of trisomy 21 [17]. The 3D ultrasound can identify an absent nasal bone in multiplanar and transparent mode. Arhinia (absence of the fetal nose) may be an isolated congenital malformation, part of aneuploidy such as trisomy 21, or involved in complex malformation such as holoprosencephaly or monogenic disease as in Treacher Collins syndrome [17]. Rather than a fetal nose, a non-functional nose in the form of a proboscis (a tubular appendix) above the central eye may sometimes exist, as in cyclopia and cebocephaly, which can occur in 1 in 16,000 and 1 in 40,000 births, respectively [18].

### 1.4. Tongue

Macroglossia is rare condition indicative of tongue hypertrophy. It can be a single sporadic trait, a family trait, or in association with Beckwith–Wiedemann syndrome or Down syndrome. According to Simmonds, congenital macroglossia is present in approximately 4.63 cases for 100,000 births, with 48.1% of cases being isolated and 51.9% being syndromic [19].

### 1.5. Lip and Palate

**Figure 1:**
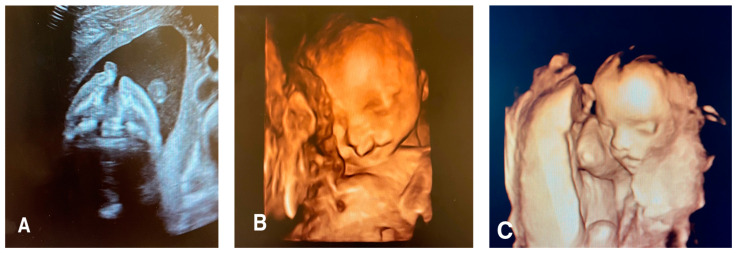
(**A**) Axial view of a 21 week old fetus with unilateral left cleft lip, cleft palate, and premaxillary protrusion (**B**) A 3D surface rendered image of the same fetus with unilateral left cleft lip and cleft palate. (**C**) A 3D surface rendered image of a 16 week old fetus with isolated left unilateral cleft lip.

Facial clefts include a wide variety of pathologies that result from the failure of fusion in the facial area during the early embryonic/fetal period, leading to a gap in the fetal face [20]. These clefts can affect the lip, philtrum, alveolus, and hard and soft palate to varying degrees. Facial clefts may be typical or atypical (Figure 1).

#### 1.5.1. Typical Forms

These include LS (isolated cleft lip), PS (isolated cleft palate), and LPS (cleft lip + isolated cleft palate). LPS represents 50% of cases, while LS and PS represent 25% each. LS incidence is higher among males than females with a 2:1 ratio, whereas PS incidence is higher among females than males with a 2:1 ratio [21]. A study conducted in Norway showed that LPS was unilateral in 64% of cases and bilateral in 33% of cases, with 3% in the midline. The same study showed that 69% of the clefts affected the left side, while 31% affected the right side. All types of clefts may also be isolated, associated with other structural anomalies, or be part of genetic syndromes or chromosomopathies. Offerdal and their team, in 2008, demonstrated that LPS was present in 43% of cases, and PS was present in 58% of cases where an association with other structural abnormalities was present. Indeed, studies have shown that orofacial clefts can be associated with several factors, including specific medications and exposure to environmental elements such as agricultural solvents. Phenobarbital, phenytoin, and diazepam during pregnancy are associated with increased incidence of orofacial clefts [22]. Use of folic acid supplements may partially prevent cleft lip and palate [23].

#### 1.5.2. Atypical Forms

These include median and lateral forms and represent 3% of cases. Median clefts are sometimes associated with holoprosencephaly or fronto-nasal dysplasia [24]. Lateral forms are characterized by an enlargement of the oral commissure, extending to the external ear, maxilla, and ascending branch of the mandible [25]. During the second trimester, the most effective way to visualize orofacial clefts is through the coronal and axial planes. A 3D ultrasound can also be used to observe these malformations. The axial plane allows evaluation of the involvement of the alveolus or primary palate and assesses possible extension to the secondary or hard palate. However, the detection rate of lateral clefts and lateral palatal clefts can vary significantly, ranging from 9% to 100% in multi-center studies. Isolated palatal clefts have a narrower detection rate interval, varying from 0% to 22%. The sagittal plane may reveal a protrusion of the palate and lip in lateral palatal clefts but is less useful in isolated lateral clefts and palatal clefts [26].

### 1.6. Epignathus

Epignathus is a solid tumor originating from the sphenoid bone, hard and soft palate, pharynx, tongue, or jaw [27]. The tumor grows into the oral or nasal cavity or intracranially. Differential diagnosis includes teratomas of the neck, encephaloceles, and other tumors of the facial structures. Polyhydramnios is generally present as a result of pharyngeal compression. This tumor can fill the mouth and respiratory tract, leading to acute asphyxia immediately after birth. Diagnosis is not always possible in the second trimester, as these tumors sometimes appear later during pregnancy [28].

### 1.7. Mandible

The most common fetal anomalies of the fetal mandible are described in this section.

#### 1.7.1. Otocephaly

Otocephaly is a rare anomaly characterized by the association of the absence or hypoplasia of the mandible, agnathia (agenesis of mandible or mandibular hypoplasia), melotia (abnormal horizontal position of the ears), microstomia (small mouth), and aglossia or microglossia (absent or rudimentary tongue). Otocephaly can be included among very severe malformation complexes, such as joint twins and holoprosencephaly. Diagnostic suspicion occurs when the jaw is not visualized and the ears are noted in a very low position. Differential diagnosis during prenatal ultrasound should be carried out under other conditions characterized by very low-set ears, such as Treacher Collins syndrome [29].

#### 1.7.2. Micrognathia/Retrognathia or Prognathia

**Figure 2:**
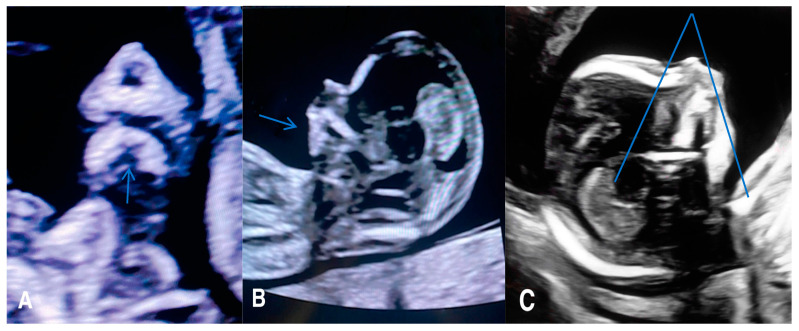
(**A**) A 2D coronal scan of the postnasal triangle of a 12 week old fetus where absence of the regular gap (see arrow) within the mandible indicates micrognathia and/or retrognathia. (**B**) Axial scan of the same fetus where the arrow indicating underdevelopment and/or posterior displacement of the chin (micrognathia and/or retrognathia). (**C**) Axial scan of the same fetus of 18 weeks with an inferior facial angle (IFA) (blue lines) of 45 degrees, diagnostic of micrognathia and/or retrognathia.

Micrognathia indicates a small chin secondary to underdevelopment of the mandible, while retrognathia means as abnormal posterior placement of the mandible (Figure 2).

These two findings pose challenges in differentiation and can manifest concurrently. Micrognathia is frequently linked to syndromes or other abnormalities. Notable syndromes associated with micrognathia/retrognathia include Treacher Collins, Pierre Robin sequence, Stickler, and 22q11.2 deletion syndrome. Ultrasound imaging plays a crucial role, particularly the mid-sagittal profile, which helps evaluate the abnormal jaw position.

Numerous quantitative methods are considered for diagnosing micrognathia, utilizing both 2D and 3D ultrasound. Rotten et al. defined two-dimensional and three-dimensional sonographic parameters to objectively diagnose retrognathia and micrognathia between 18 and 28 weeks of gestation. Their findings indicated that retrognathia is associated with a decrease in the inferior facial angle (IFA). Therefore, a diagnosis of micrognathia/retrognathia can be established when the IFA measures less than 49.2°. This angle is defined by an orthogonal line to the vertical section of the forehead at the level of the nasal bone synostoses and a line connecting the chin’s tip to the anterior limit of the protrusive margin [30]. Despite these developments, diagnosing micrognathia/retrognathia remains challenging during both the second and the first trimesters. To address this, Sepulveda et al. proposed using ultrasound scans in the coronal view of the retronasal triangle to assess the midface during the first trimester. Their study revealed that a normal fetus exhibits a gap between the two mandibular bones, referred to as the mandibular gap. In contrast, fetuses with micrognathia/retrognathia lack this gap, or the mandible cannot be identified at this level. However, further research is necessary to determine the sensitivity and specificity of the first-trimester retronasal triangle view to detect cleft palate. Consequently, an early-second-trimester targeted scan remains a complementary diagnostic examination to consider [31].

### 1.8. Craniosynostosis

Craniosynostosis is a condition characterized by the premature closure of one or more cranial sutures. It occurs at a rate of 4.3 cases per 10,000 births, with approximately 85% of cases manifesting in an isolated form, while the remaining 15% are associated with syndromes or other anomalies [32]. Among single suture synostoses, the sagittal suture is the most commonly affected, followed by the coronal, metopic, and lambdoidal sutures, each influencing the shape of the skull accordingly.

A less common variant of craniosynostosis is the Kleeblattschaedel type, involving the fusion of multiple sutures, resulting in a clover-shaped appearance of the skull. Syndromic craniosynostoses are more severe and often present in conjunction with other anomalies, such as those seen in Apert, Peiffer, Crouzon, Jackson–Weiss, and Carpenter syndrome [33]. Detecting craniosynostoses via ultrasound is challenging, with typically only the most severe cases being diagnosed. However, when biometric abnormalities are observed in the biparietal and occipitofrontal diameter of the fetal head, it should prompt diagnostic suspicion. In such instances, magnetic resonance imaging (MRI) serves as a valuable complementary tool to support ultrasound findings [34].

## 2. Materials and Methods

**Figure 3:**
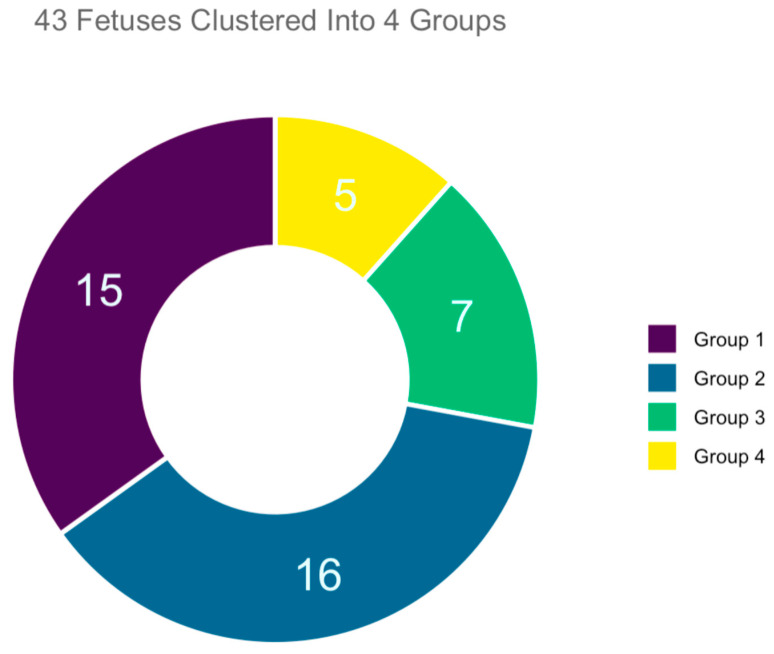
Representation of four main groups identified by autopsy in a sample of 43 fetuses with facial defects. The legend on the right-hand side corresponds to fetal groups. On the basis of the autopsy fetal analysis, Group 1 (15/43) contained fetuses with facial anomalies.

This was a retrospective review of 43 autopsies of fetuses with facial anomalies performed at a third-level university hospital and the corresponding prenatal ultrasound examination performed at the same center in the period between January in 2010 and January 2020. During this 10-year interval, a total of 300 autopsies of fetuses with malformation were performed, followed intrauterine death or voluntary termination of pregnancy after 13 weeks of pregnancy. For purposes of this research study, we only considered those with facial anomalies, specifically 53 fetuses. Of these, 10 had to be excluded because the corresponding prenatal ultrasound documentation were no longer available. The total number of cases accepted and enrolled in the study was 43 (Figure 3). All the cases enrolled had undergone a first-trimester combined screening for trisomies; in all cases, regardless of the calculated risk of trisomies, US findings suggested possible anatomical anomalies. Thus, a targeted early-second-trimester ultrasound scanning was performed for a more accurate prenatal diagnosis.

Karyotyping was performed in all 43 cases enrolled. Depending on the week of gestation at the time of investigation, the invasive prenatal diagnosis technique chosen was amniocentesis, between the 15th and 20th weeks, or chorion villous sampling (CVS), between the 10th and 12th weeks. This investigation allowed categorizing and distinguishing between isolated or associated facial anomalies and concomitant chromosomal or genetic disorders.

Obtaining obstetric ultrasound data was obviously a key element for the realization of the project; however, its success is based on the possibility of comparing prenatal diagnostic data with autopsy data. It was, therefore, crucial to make the investigation anonymous and “confidential”, without giving rise to the suspicion that it hides inquisitorial or judgmental purposes of the individual operators of our operating unit. The proposed study was achievable because it was based on diagnostic work already conducted in the prenatal and autopsy domains. Fetal autopsy diagnostics were carried out by highly skilled pathologists of Bari Polyclinic, following a standardized procedural protocol for conducting such autopsies. Additionally, there was seamless collaboration among the designated pathologists during the fetus analysis process, fostering a collective approach to the assessments. Their data were stored in electronic reporting formats, ensuring effortless traceability. The Gynecology and Obstetrics section had archived the reporting data in paper format; therefore, the medical records were recovered, and the data were computerized in homogeneous manner. Among the critical points of the project, the execution of the autopsy for diagnostic confirmation is mandatory in Italy only for the “stillborn” (Legislative Decree 9 July 1999—Official Journal general series 170 of 22 July 1999), i.e., for fetuses born after 25 weeks of pregnancy. The autopsy of fetuses from TOP, although not subject to parental authorization, remains a decision of the attending physician. In the present study, this criticality was overcome since, in our structure, the diagnostic verification of TOP fetuses is traditionally carried out at least in cases where there is suspicion of malformative pathologies. Advanced maceration or fragmentation due to hysterosuction was an exclusion criterion; therefore, only fetuses that received the autopsy examination in autopsy conditions were enrolled. Specifically, ultrasound and/or autopsy agreement analysis was divided into five categories:

- A1—complete agreement of ultrasound with autopsy,
- A2—autopsy confirms but adds details undetectable with ultrasound,
- B—autopsy confirms but adds details detectable with ultrasound,
- C—ultrasound findings partially demonstrated by autopsy,
- D—total discrepancy between ultrasound and autopsy and/or non-identification.

The final count of cases accepted in our research study was 43, with gender granularity of 20 females and 23 males. Their maternal age ranged from 14 to 46, with a mean age of 32 years. Within our group, 21/43 were nulliparous, and 7/43 were in vitro fertilization pregnancies. Another key quantity to mention here is the week of gestation (WG). At the time of ultrasound diagnosis, the range was between 13 and 25 WG, with a mean value of 21 weeks. The gestational week of fetuses analyzed after IUD or TOP ranged between 13 and 31 weeks.

## 3. Results

From analyzing the autopsy reports and performing clustering analysis, we identified 15/43 cases where fetuses with facial malformations were not associated with syndromes or numerical alterations of chromosomes (clustered into Group 1). Next, we had 16/43 fetuses with facial malformations and other associated organ anomalies, not related to syndromes or numerical alterations of chromosomes (clustered into Group 2), while 7/43 cases were fetuses with facial malformations and prenatal detection of partial deletions of chromosomes, chromosomal structural anomalies, or chromosomal mosaicisms (clustered into Group 3). Lastly we had a cluster of 5/43 fetuses with facial malformations and prenatal recognition of a numerical chromosomal disorder (clustered into Group 4).

Firstly, Group 1 represented 15/43 of overall cases in selected data sample. Within this group, we identified 6 cases of cleft lip and palate, 3 cases of cleft lip, 2 cases of cleft palate, 1 case of microphthalmia, 2 cases of micrognathia, and 1 case of craniosynostosis. Secondly, Group 2 contained 16 cases, specifically, 5 cases of cleft lip and palate, 4 cases of cleft palate, 3 cases of cleft lip, 2 cases of microphthalmia, and 2 cases of micrognathia. It is important to mention here that 9 cases of 16 of facial anomalies in Group 2 were associated with the cardiovascular system.

**Figure 4:**
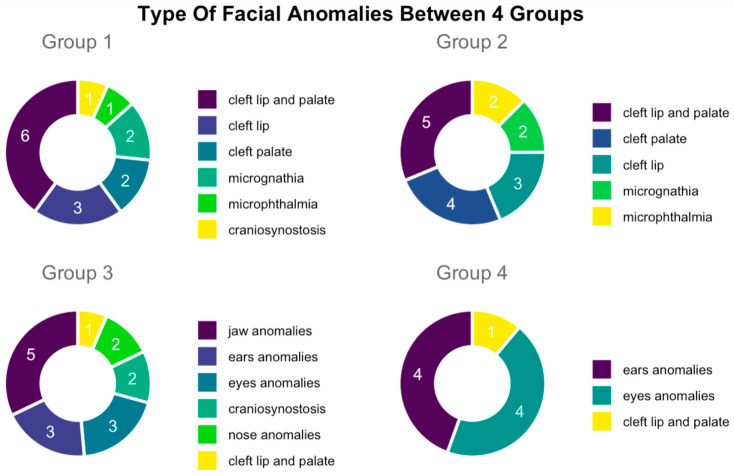
Number of cases per group with specific facial anomaly.

Secondly, 8/16 cases were associated with the urinary system, 5/16 were associated with the central nervous system, 3/16 were associated with anomalies of the skeletal system, and 2/16 were associated associated with the digestive system. Thirdly, Group 3 represented 7/43 fetuses with one each of seven syndromes—3M syndrome, Pierre Robin sequence (PRS), Pfeiffer syndrome, Binder syndrome, Freeman–Sheldon syndrome, Crouzon, and Treacher Collins syndrome. Among these facial defects that characterized this group, 5 of 7 were abnormalities of the mandible, 2 of 7 cases were cleft lip and palate abnormalities, and 1 case was of macroglossia. Lastly, Group 4 consisted of 5/43 cases, with 1 case of trisomy 21 and 4 cases of trisomy 13. In particular, facial anomalies in this group involved the ears and eyes in 4/5 cases, whereas 1/5 had a cleft lip and palate (Figure 4).

**Figure 5:**
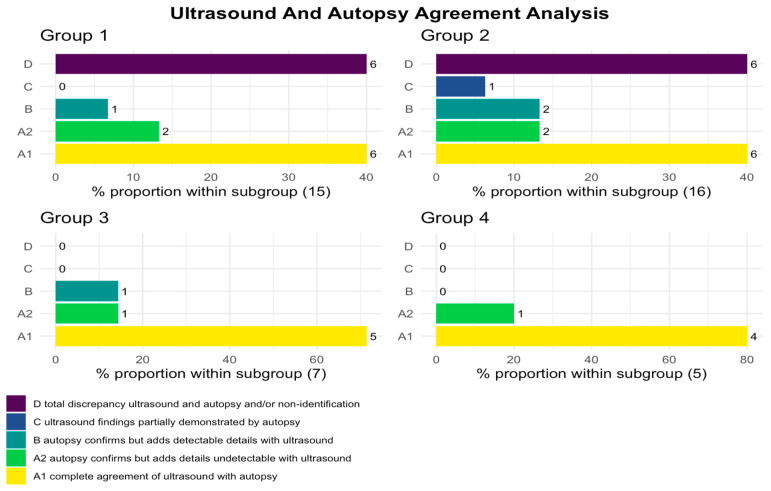
Ultrasound and autopsy agreement analysis. Number of cases per group with assigned Category (A1, A2, B, C, and D). Ultrasound diagnosis for Group 1 and Group 2 (Categories A1 + A2) showed low agreement rate with autopsy reports. Ultrasound diagnosis for Group 3 and Group 4 (Categories A1 + A2) showed high concordance with autopsy examination.

A thorough comparison of the facial defects detected by ultrasound and autopsy revealed that, in Group 1 and Group 2, ultrasound diagnosis showed a low agreement rate with autopsy reports. Only 53.3% (8/15) cases in Category A1 and in Category A2 had concordance with autopsy examination; moreover, 40% (6/15) cases in Category D were not recognized or were discordant with the autopsy examination. Similar concordances and matching were found in Group 2, where 43.75% (7/16) (Category A1 and Category A2) had concordance, while 37.5% (6/16) (Category D) were not recognized or were discordant from autopsy examination. In contrast, Group 3 had an agreement rate of 85.7% (6/7) (Category A1 and Category A2) with the autopsy examination, whereas no cases (0/7) (Category D) were not recognized or were discordant with the autopsy examination. Similar agreements were found in Group 4 where 100% (5/5) (Category A1 and Category A2) had agreement with the autopsy examination, whereas no cases (0/7) (Category D) were not recognized or were discordant with the autopsy examination (Figure 5).

Our study further showed that facial anomalies were predominant in Group 1 and Group 2 (31/43), compared to Group 3 and in Group 4 (12/43 in total; 7/43 and 5/43, respectively). Specifically, in Group 1 and Group 2, lip and palate anomalies predominated, specifically, 73.3% of cases (11/15) in Group 1 and 75% of cases (12/16) in Group 2. In Group 3, however, abnormalities of the mandible 71.4% (5/7) predominated. In Group 4, fetal eyes and fetal ears represented the same proportion, both 80% (4/5). In Group 2 of fetuses with facial malformations associated with other organ anomalies, the distribution of malformations by system mostly involved the heart and great vessels. These were represented by 56.2% (9 of 16) of cases, followed by those of the genitourinary system at 50% (8 of 16), of the central nervous system at 31.2% (5 of 16), of the skeletal system at 18.7% (3/16), and of the digestive system at 12.5% (2/16).

## 4. Discussion

Retrospectively, we clustered and analyzed anatomopathological characteristics on a sample of 43 fetuses with facial anomalies of low gestational age of women following intrauterine death or legal termination of pregnancy during the years 2010–2020. This type of study is the first of its kind in Italy. Rigorous comparison of prenatal ultrasound diagnosis of the malformation pattern and the subsequent autopsy led the physician to a self-critical analysis of their diagnosis with improvement of their future diagnostic skills. Since both compared methods had specific limitations (listed below), adding comprehensive insights from both can be beneficial for a physician.

Both methods, prenatal ultrasound and autopsy, come with critical issues to account for. Firstly, the prenatal ultrasound investigation of a fetus represents an indirect type of diagnosis, burdened by all the factors that modulate the transmission of ultrasound from probe to fetus and then reflecting from fetus to probe. In this way, the ultrasound crosses the fetal tissues, as well as the amniotic fluid, uterine wall, and adipose, muscle, and skin layers of women. Additionally, increased levels of amniotic fluid (polyhydramnios), or its strong reduction (oligohydramnios) or absence (anhydramnios), are conditions that can greatly affect the diagnostic accuracy of prenatal ultrasound evaluation. On the other hand, the autopsy examination requires special measures such as an expert team of anatomopathologists, a suitable dissecting photomicroscope, and a short time-lapse between TOP or IUD and autopsy. Autoptic findings made on fetuses of low gestational age may not manifest of clearly show malformations at the time of the examination (ultrasound as autopsy).

Despite listed limitations for both methods, our study revealed a high level of agreement between prenatal ultrasound and autopsy findings in the groups (Group 3 and Group 4) of fetal facial abnormalities associated with genetic syndrome or numerical abnormality of chromosomes. The main reason is that prenatal recognition of chromosome anomalies and genetic syndromes requires the scan operator to search for a specific malformation pattern and, therefore, to correct ultrasound diagnosis. On the other hand, our study manifested a lower level of agreement between prenatal ultrasound and autopsy findings in the groups (Group 1 and Group 2) with isolated facial defects or those associated with other organ anomalies, but not associated with genetic syndrome or numerical chromosome anomaly.

Summarizing the results of our study, the importance of a targeted ultrasound study of the facial anatomy can be highlighted, which should, indeed, be offered to all women at the beginning of the second trimester. As a matter of fact, a routine execution of early second-trimester examination would refine the operator-dependent diagnostic accuracy of ultrasound scan, which, as demonstrated in our study, seems to be reduced in cases of facial anomalies not linked to genetic disorders. Moreover, although the recent ISUOG guidelines do not yet expressly indicate this, our study showed that the use of 3D and 4D techniques has made it easier for operators to refine diagnostic accuracy, especially in minor defects. In terms of prognosis quod vitam, the high ultrasound detection rate of facial anomalies in groups with genetic anomalies represents a crucial additional cognitive element in the parental decision-making process. Conversely, the reduced sensitivity in the diagnosis of non-genetically related facial anomalies could sometimes overlook isolated cases of malformations with a reduced quod vitam impact on the unborn child, thus not entirely disabling the reliability of the ultrasound examination itself. Most studies reported different detection rates of facial defects, but the results varied considerably by population (high or low risk), type of defect, and gestational age at the time of examination. Lai et al. showed that fetal ultrasound in the second and third trimesters has excellent sensitivity and specificity to detect cleft palate in high-risk pregnancies [35]. Deng et al. highlighted how three-dimensional ultrasound can significantly improve the diagnostic accuracy of prenatal cleft palate. In their study, the two-dimensional ultrasound cleft palate detection rate was 36.8% compared to 89.5% for three-dimensional ultrasound cleft palate detection [36]. Farladansky-Gershnabel et al. showed in their study that isolated cleft lip was diagnosed in 5/7 cases (71.5%), along with combined clefts in 29/38 cases (76.3%) and CP in 7/51 cases (13.8%) [37]. The 2D and 3D ultrasound scans have the same accuracy for cleft lip. The 3D ultrasound should be used for a secondary assessment following 2D ultrasound if there is a suspicion of cleft lip [38].

Similarly, Mouthon et al. demonstrated through a retrospective analysis on 41 fetuses affected by micrognathia that their prenatal detection rate was 29%, lower than that reported by other previous studies [39].

The prenatal ultrasound detection rate of craniosynostosis is low. Harada et al. investigated 41 cases of craniosynostosis, and the prenatal ultrasound detection rate of these was 61% [40]. Research conducted on different fetal anatomical regions highlights the necessity for specialized cohort studies incorporating multivariate analysis. These studies aim to assess the prognostic significance of cranial ultrasound and determine the appropriate timing and necessity of its screening protocol [41].

Prenatal diagnosis of microphthalmia and anophthalmia is sometimes not straightforward and requires high-quality ultrasonography. Existing prenatal ultrasound protocols for fetal eye imaging are inconsistent and inadequate to detect the specificity of the ocular malformation, and there are no clear guidelines for detecting such rare abnormalities [42].

## 5. Conclusions

Prenatal diagnosis of craniofacial anomalies remains difficult, especially in the first trimester. A systematic approach to the fetal face and skull using additional ultrasound scan planes such as sagittal for the profile, coronal for lips and nares, and axial for alveolar arch and palate, along with a 3D method and more advanced training for clinicians and professionals, can greatly improve the sensitivity of the overall diagnosis. Ultimately, if the ultrasound is essential for prenatal screening of fetal facial defects, as well as many other pathologies, and the autopsy is the most suitable tool for the diagnostic definition of these, the importance of their integration emerges for an epicritical reconstruction of the pathological picture observed. Furthermore, the operator might improve their own diagnostic skills by performing a regular comparison with the autopsy examination reports.

## Data Availability

All results are reported within the text.
